# Drug use among youth and adults in a population-based survey in South Africa

**DOI:** 10.4102/sajpsychiatry.v24i0.1139

**Published:** 2018-04-12

**Authors:** Karl Peltzer, Nancy Phaswana-Mafuya

**Affiliations:** 1 HIV/AIDS/STIs and TB Research Programme, Human Sciences Research Council, South Africa; 2 Department of Research Innovation and Development, University of Limpopo, South Africa; 3 Deputy Vice Chancellor Research and Innovation Office, North West University, South Africa

## Abstract

**Objective:**

Illicit drug use is a growing public health problem. The aim of the study was to assess the prevalence of drug use and the sociodemographic and health characteristics that influence it among young and adult South Africans.

**Methods:**

Data based on the South African national population-based survey in 2012 for 26 453 individuals (52.0% women and 48.0% men) aged 15 years and older were analysed. Past 3-month drug use was assessed with the ‘Alcohol, Smoking and Substance use Involvement Screening Test (ASSIST)’. Bivariate and multivariable logistic regression was conducted to assess the association between sociodemographic factors, health variables and any past 3-month drug use.

**Results:**

Overall, any past 3-month drug use was 4.4%, 7.9% among men and 1.3% among women. The proportion of past 3-month cannabis use was 4.0%, followed by sedatives or sleeping pills 0.4%, amphetamine-type stimulants 0.3%, cocaine 0.3%, opiates 0.3%, inhalants 0.2% and hallucinogens 0.1%. Among the nine South African provinces, any past 3-month drug use was the highest in the Western Cape (7.1%), followed by the Free State (6.3%) and Northern Cape (5.2%). In adjusted, multivariable, logistic regression analysis among both men and women, younger age, being mixed race and hazardous or harmful alcohol use were associated with any past 3-month drug use. In addition, having been a victim of violent crime and sexual risk behaviour among men and having psychological distress among women were associated with any past 3-month drug use.

**Conclusion:**

An increase of any past 3-month drug use from 3.7% in 2008 to 4.4% in 2012 was observed in South Africa. Prevention and intervention activities targeting drug use, in particular in identified risk groups, need to be strengthened in South Africa.

## Introduction

Illicit drug use is a growing public health problem. The estimated global prevalence of illicit drug use (including amphetamines, cannabis, cocaine, opioids, etc.) is 5.3% in the past year in 2014.^1,2^ The most commonly used illicit drugs globally are cannabis, amphetamine-type stimulants, cocaine and opioids.^2^ Illicit drug use contributes significantly to the global burden of disease, that is 0.8% in 2010.^3^

In a US population-based survey conducted among individuals aged 12 years and older in 2015, past 1-month (any) illicit drug use was 10.1%.^4^ In a South African population-based national study conducted in 2008, the prevalence of past 3-month (any) drug use was 3.7%.^5^ The highest past 3-month prevalence was for cannabis (3.3%), followed by sedatives or sleeping pills (0.8%), amphetamine-type stimulants (0.7%), cocaine (0.6%), opiates (0.5%) and hallucinogens (0.5%).^5^

Previous investigations in South Africa and other countries found that specific sociodemographic factors were associated with drug use, including male gender,^5,6^ younger age,^5^ specific population groups (mixed race and white people),^5,6^ lower income or not employed^5^ and geolocality such as urban areas.^5,7^ Further, certain health risk behaviours such as common mental disorders (major depression and anxiety disorders),^8,9^ alcohol use disorders,^10^ HIV risk behaviours^11^ and criminal victimisation^12^ have been found to be associated with drug use.

In order to update planning on drug use programming, more recent national population-based prevalence data on illicit drug use among adolescents and adults in South Africa are needed. Therefore, the purpose of this secondary analysis was to make more recent estimates on the frequency and type of drug use among women and men 15 years and older available using a nationally representative household survey in 2012.

## Method

### Data and sampling

Cross-sectional data from the ‘2012 South African national HIV prevalence, incidence, and behaviour survey’ was analysed.^13^ The sampling strategy was stratified by province, type of geolocality and predominant population or racial groups. Using multistage sampling, a random sample of ‘enumeration areas’ was selected, and within enumeration areas households were randomly selected. All individuals within a household were eligible to participate. Trained and supervised field workers interview-administered a questionnaire. Informed consent was attained prior to the conduct of the interview. The detailed survey methods are described elsewhere.^13^

#### Measures

*Drug use* in the past 3 months was assessed with 10 items of the ‘Alcohol, Smoking and Substance Involvement Screening Test (ASSIST)’, e.g. ‘In the past 3 months, how often have you used cannabis (dagga, marijuana, pot, grass, hash, etc.)?’^14^ This included the item ‘Besides drugs prescribed by a health professional, have you ever used a drug by injection?’ The response option was ‘never’, ‘Yes. In the past 3 months’ and ‘Yes, but not in the past 3 months’. One item was added ‘Whoonga (mixture of heroin, dagga = cannabis and antiretrovirals)’.^13^ Response options ranged from 1 = never to 5 = almost daily. Any drug use in the past 3 months was coded as 1 and never as 0. All items were added together to indicate the prevalence of any illicit drug use in the past 3 months. Cronbach’s alpha for this section of the ASSIST in this sample was 0.61.

**Demographic measures** included age, educational level, race (African black people or other races), geolocality, province and employment status.

**Psychological distress** was measured with the ‘Kessler Psychological Distress Scale (K-10)’.^15^ The 10-item scale assesses global psychological distress experienced in the past 30 days, e.g. ‘In the past 30 days, how often did you feel so restless that you could not sit still?’ Response options ranged from 1 = ‘none of the time’ to 5 = ‘all the time’. These scores were added up, with higher total scores indicating higher psychological distress.^15^ A cut-off of 16 scores and more for detecting depression and anxiety disorders was used, as suggested in a previous validation study of the K-10 in the general population in South Africa.^16^ Cronbach’s alpha for the K-10 in this sample was 0.89.

**Hazardous or harmful alcohol use** was assessed with the 10-item ‘Alcohol Disorder Identification Test (AUDIT)’, e.g. ‘How often did you have a drink containing alcohol in the past 12 months?’^17^ Response options ranged from 0 to 4, with a summed total range from 0 to 40 scores; a score of 8 or more indicated hazardous or harmful or probable dependent drinking.^17^ Cronbach’s alpha for the AUDIT in this sample was 0.84.

**Violent crime victimisation** was assessed with the question, ‘In the past 12 months, have you been a victim of a violent crime where a gun or knife was used to threaten or harm you?’ Response option was ‘yes’ or ‘no’.^13^

**Sexual risk behaviour** was assessed with two questions. (1) ‘Have you had sex during the past 12 months?’ (2) ‘Overall, how many sexual partners did you have during the past 12 months?’ (coded two or more = 1 and 0 to 1 sexual partner = 0).^13^

#### Data analysis

Descriptive statistics were used to summarise drug use prevalence, sociodemographic factors and health variables. Associations between the outcome variable of any past 3-month drug use (cannabis, cocaine, amphetamine, inhalants, sedatives, hallucinogens or opiates including *Whoonga*), sociodemographic and health risk-independent variables were examined by calculating odds ratios. Unconditional multivariable logistic regression was utilised to assess the impact of explanatory variables for the outcome of past 3-month drug use prevalence, for women and men separately. All variables that were statistically significant at the *p* < 0.05 levels in bivariate analyses were included in the multivariable models. In the article, weighted percentages are presented. The ‘svy’ command was utilised to take into account the multistage cluster design of the survey. All statistical analyses were performed by using Stata software version 12 (Stata Corp., College Station, TX, USA).

## Ethical consideration

This analysis is based on data on individuals aged 15 years and older who participated in the survey. The study survey proposal was approved by the ‘HSRC Research Ethics Committee (REC: 5/17/11/10)’ and by the ‘Centers for Disease Control and Prevention’ (CDC).

## Results

### Sample characteristics and prevalence of drug use

Response rates for the interview was 89.5%.^13^ The total sample included 26 453 persons aged 15 years and older, 52.0% women and 48.0% men. More than half of the participants (52.0%) were between 15 and 34 years old, 40.2% had Grade 12 or more education, 77.8% were from the African black population group, 39.3% were formally or informally employed and 51.9% lived in urban formal areas. A large proportion of the participants (37.8%) reported psychological distress, 11.1% hazardous or harmful or probable dependent alcohol use, 14.7% had become victim of a violent crime in the past 12 months and 19.1% had more than one sexual partner in the past 12 months.

Overall, the past 3-month of any drug use was 4.4%, 7.9% among men and 1.3% among women. The proportion of the past 3-month cannabis use was 4.0%, followed by ‘sedatives or sleeping pills (Valium, Mandrax, Serepax, Rohypnol, etc.)’ 0.4%, amphetamine-type stimulants (speed, ecstasy, tik, etc.) 0.3%, ‘cocaine (coke, rocks, crack, etc.)’ 0.3%, ‘opiates (heroin, morphine, methadone, codeine, *Whoonga* [mixture of heroin, cannabis and alleged antiretrovirals], etc.)’ 0.3%, ‘inhalants (nitrates, glue, petrol, paint, thinners, etc.)’ 0.2%, and ‘hallucinogens (lysergic acid diethylamide [LSD], acid, mushrooms, Phencyclidine [PCP], Special K, etc.)’ 0.1%. The prevalence of past 3-month drug by injection was 0.6%, and the use of *Whoonga* 0.2%. Among the nine South African provinces, any past 3-month drug use was the highest in the Western Cape (7.1%), followed by the Free State (6.3%), Northern Cape (5.2%) and Gauteng (4.9%) (see Table 1).

**TABLE 1:** Past 3-month prevalence of drug use (*N* = 26 453).

Variable	Any drug use			Cannabis	Cocaine	Amphetamine	Inhalants	Sedatives	Hallucinogens	Opiates†

	Total *N* (%)	Men (%)	Women (%)	Total (%)	Total (%)	Total (%)	Total (%)	Total (%)	Total (%)	Total (%)
**Sociodemographics**										
All	1092 (4.4)‡	7.9	1.3	4.0	0.3	0.3	0.2	0.4	0.1	0.3
**Age**										
15–24 (27.6%)	422 (5.7)	9.6	1.8	5.6	0.4	0.4	0.2	0.3	0.3	0.4
25–34 (24.4%)	316 (6.5)	12.0	1.0	6.1	0.5	0.4	0.3	0.4	0.1	0.5
35–44 (19.1%)	168 (3.8)	6.3	1.4	3.2	0.3	0.4	0.2	0.5	0.0	0.2
45–54 (13.1%)	93 (2.3)	3.7	1.1	1.8	0.2	0.1	0.0	0.4	0.0	0.1
55 or more (15.7%)	92 (1.5)	2.4	1.0	0.9	0.2	0.1	0.1	0.5	0.0	0.0
**Education**										
Grade 0–7 (18.0%)	185 (4.6)	8.4	0.8	4.2	0.4	0.3	0.2	0.5	0.1	0.2
Grade 8–11 (41.8%)	451 (4.9)	8.9	1.2	4.5	0.3	0.3	0.2	0.2	0.1	0.4
Grade 12 or more (40.2%)	320 (4.1)	6.7	1.6	3.8	0.3	0.3	0.1	0.6	0.2	0.2
**Population group**										
African black people (77.8%)	514 (4.0)	7.5	0.7	3.7	0.2	0.2	0.2	0.2	0.1	0.2
White people (10.1%)	114 (5.0)	6.0	4.0	4.2	0.8	0.6	0.2	1.2	0.1	0.1
Mixed race (9.3%)	361 (8.3)	13.7	3.4	7.4	0.6	1.1	0.4	1.0	0.5	0.9
Indian or Asian (2.8%)	102 (3.2)	4.8	1.7	2.8	0.4	0.3	0.0	1.1	0.2	0.1
**Employment status**										
Employed (39.3%)	475 (5.1)	7.4	1.9	4.6	0.3	0.3	0.1	0.5	0.3	0.3
Unemployed¶ (27.4%)	337 (6.1)	12.7	1.1	5.6	0.5	0.4	0.3	0.4	0.0	0.5
Student (16.8%)	132 (3.5)	5.6	1.4	3.4	0.2	0.3	0.2	0.2	0.1	0.2
Unemployed§ (13.7%)	59 (1.7)	4.9	0.7	1.3	0.3	0.2	0.2	0.3	0.1	0.1
Unable to work (2.8%)	21 (4.4)	7.2	0.2	4.3	0.3	0.0	0.0	0.1	0.0	0.3
**Residence**										
Urban formal (51.9%)	743 (5.5)	9.3	2.0	5.0	0.5	0.5	0.2	0.7	0.2	0.4
Urban informal (7.8%)	101 (4.1)	7.7	0.4	3.9	0.1	0.0	0.0	0.2	0.0	0.2
Rural informal (34.9%)	126 (2.8)	5.7	0.4	2.5	0.2	0.1	0.1	0.0	0.0	0.1
Rural formal (5.4%)	122 (5.0)	7.5	1.7	4.9	0.3	0.3	0.2	0.2	0.1	0.5
**Province**										
Western Cape (12.2%)	248 (7.1)	11.6	2.9	6.4	0.4	0.8	0.1	0.9	0.1	0.3
Eastern Cape (11.9%)	123 (4.1)	7.3	0.7	3.9	0.2	0.1	0.2	0.3	0.1	0.1
Northern Cape (2.2%)	94 (5.2)	9.3	1.3	4.8	0.2	0.8	0.3	0.4	0.2	0.6
Free State (5.4%)	90 (6.3)	10.3	2.3	5.4	0.4	0.4	0.4	0.9	0.0	0.7
KwaZulu-Natal (18.5%)	209 (3.5)	7.0	0.5	3.4	0.1	0.2	0.0	0.2	0.0	0.1
Northwest (6.9)	48 (2.7)	5.0	0.4	2.4	0.1	0.1	0.1	0.2	0.1	0.4
Gauteng (25.5%)	175 (4.9)	8.4	1.6	4.6	0.5	0.3	0.2	0.5	0.4	0.4
Mpumalanga (7.5%)	52 (3.6)	5.6	1.4	2.9	0.1	0.3	0.1	0.1	0.0	0.3
Limpopo (9.9%)	53 (2.9)	6.1	0.5	2.2	0.7	0.1	0.3	0.1	0.1	0.1
**Health risk variables:Psychological distress (**≥**16)**										
No (62.2%)	568 (3.8)	6.4	0.9	3.5	0.3	0.2	0.1	0.3	0.1	0.2
Yes (37.8%)	495 (5.4)	10.8	1.7	4.9	0.4	0.5	0.2	0.6	0.2	0.4
**Hazardous/harmful alcohol use**										
No (88.9%)	688 (2.9)	5.3	1.1	2.6	0.2	0.2	0.1	0.3	0.1	0.2
Yes (11.1%)	400 (16.4)	18.7	5.5	15.9	1.2	1.4	0.6	0.9	0.3	1.0
**Victim of violence crime**										
No (92.1%)	927 (4.0)	7.3	1.1	3.6	0.3	0.2	0.2	0.3	0.1	0.3
Yes (7.9%	159 (9.9)	13.2	4.8	9.4	0.6	1.0	0.4	1.4	0.7	0.4
**Two or more sexual partners**										
No (87.4%)	545 (3.7)	6.4	1.3	3.4	0.3	0.2	0.1	0.4	0.1	0.2
Yes (12.6%)	216 (14.6)	16.9	4.2	14.6	1.2	1.2	0.7	0.7	0.8	1.2

† , Includes *Whoonga* = mixture of heroin, cannabis and antiretrovirals;

‡ , poly drug use (two or more drugs) is 0.5%;

¶ , not job seeking;

§ , job seeking.

### Associations with any past 3-month drug use

In adjusted, multivariable, logistic regression analysis among men, younger age, being mixed race, not living in a rural informal area, hazardous or harmful alcohol use, having been victim of a violent crime and having two or more sexual partners in the past 12 months were associated with any past 3-month drug use. In adjusted, multivariable, logistic regression analysis among women, younger age, being white people or of mixed race, not unemployed (looking for job), psychological distress and hazardous or harmful alcohol use were associated with any past 3-month drug use (see Table 2).

**TABLE 2:** Association between sociodemographics, health variables and drug use.

Sociodemographic variables	Men		Women	
	Unadjusted odds ratio (95% CI)	Adjusted odds ratio (95% CI)	Unadjusted odds ratio (95% CI)	Adjusted odds ratio (95% CI)
**Age**				
15–24	1 (Reference)	1 (Reference)	1 (Reference)	1 (Reference)
25–34	1.29 (1.01, 1.65)*	0.78 (0.57, 1.08)	0.53 (0.31, 0.92)*	0.39 (0.20, 0.73)**
35–44	0.63 (0.43, 0.92)*	0.44 (0.28, 0.67)***	0.75 (0.39, 1.43)	0.51 (0.23, 1.12)
45–54	0.37 (0.24, 0.56)***	0.27 (0.16, 0.46)***	0.59 (0.32, 1.08)	0.27 (0.11, 0.63)**
55 or more	0.23 (0.15, 0.65)***	0.19 (0.10, 0.37)***	0.53 (0.27, 1.03)	0.84 (0.29, 2.40)
**Education**				
Grade 0–7	1 (Reference)	-	1 (Reference)	-
Grade 8–11	1.07 (0.78, 1.47)	-	1.39 (0.68, 2.85)	-
Grade 12 or more	0.79 (0.53, 1.16)	-	1.95 (0.97, 3.92)	-
**Population group**				
African black people	1 (Reference)	1 (Reference)	1 (Reference)	1 (Reference)
White people	0.78 (0.48, 1.26)	1.12 (0.62, 2.01)	6.23 (3.55, 10.92)***	6.44 (2.93, 14.17)***
Mixed race	1.95 (1.49, 2.54)***	1.86 (1.30, 2.67)***	5.20 (3.01, 8.98)***	5.18 (2.62, 10.25)***
Indian or Asian	0.62 (0.42, 0.92)*	0.84 (0.55, 1.35)	2.53 (0.64, 9.99)	4.84 (0.99, 23.53)
**Employment status**				
Employed	1 (Reference)	1 (Reference)	1 (Reference)	1 (Reference)
Unemployed (not looking)	1.83 (1.41, 2.37)***	1.19 (0.80, 1.76)	0.59 (0.34, 1.02)	0.99 (0.55, 1.81)
Student	0.75 (0.51, 1.09)	0.70 (0.38, 1.28)	0.72 (0.36, 1.46)	0.73 (0.27, 1.99)
Unemployed (looking)	0.65 (0.38, 1.10)	1.01 (0.46, 2.22)	0.34 (0.17, 0.67)**	0.31 (0.12, 0.79)*
Unable to work	0.97 (0.45, 2.09)	0.43 (0.11, 1.74)	0.11 (0.21, 0.58)**	Too few cases
**Residence**				
Urban formal	1 (Reference)	1 (Reference)	1 (Reference)	1 (Reference)
Urban informal	0.82 (0.57, 1.16)	0.82 (0.53, 1.25)	0.21 (0.07, 0.59)**	0.35 (0.99, 1.25)
Rural informal	0.59 (0.44, 0.79)***	0.67 (0.45, 0.98)*	0.18 (0.08, 0.41)***	0.39 (0.12, 1.29)
Rural formal	0.80 (0.47, 1.35)	0.66 (0.35, 1.22)	0.82 (0.41, 1.66)	0.85 (0.32, 2.26)
**Health variables: Psychological distress (**≥ **16)**				
No	1 (Reference)	1 (Reference)	1 (Reference)	1 (Reference)
Yes	1.77 (1.30, 2.27)***	1.30 (0.96, 1.76)	1.77 (1.15, 2.73)**	2.05 (1.21, 3.47)**
**Hazardous or harmful alcohol use**				
No	1 (Reference)	1 (Reference)	1 (Reference)	1 (Reference)
Yes	4.10 (3.20, 5.27)***	2.33 (1.73, 3.15)***	5.27 (2.95, 9.44)***	3.14 (1.62, 6.09)***
**Victim of violence crime**				
No	1 (Reference)	1 (Reference)	1 (Reference)	1 (Reference)
Yes	1.95 (1.36, 2.80)**	1.65 (1.04, 2.61)*	4.77 (1.60, 4.13)***	1.88 (0.97, 3.66)
**Two or more sexual partners**				
No	1 (Reference)	1 (Reference)	1 (Reference)	1 (Reference)
Yes	2.97 (2.14, 4.12)***	1.78 (1.21, 2.61)**	3.20 (1.57, 6.50)***	1.69 (0.58, 4.87)

CI, confidence interval.

* *p* < 0.05;

** *p* < 0.01;

*** *p* < 0.001

## Discussion

In this very large national population-based study of 2012 among individuals 15 years or older found that any past 3-month drug use was 4.4%, which was higher than in the previous 2008 national survey, with 3.7%^5^ but lower than in a survey in the US (past month 10.1%).^4^ Among the different drugs used, the highest past 3-month prevalence was for cannabis (4.0%), as found in the 2008 South Africa survey (3.3%)^5^ and also in other countries such as the US.^4^ Compared to the 2008 South Africa national survey,^5^ in this 2012 survey the prevalence of past 3-month use increased from 3.3% to 4.0% for cannabis use, while it decreased for sedatives or sleeping pills from 0.8% to 0.4%, amphetamine-type stimulants 0.7% to 0.3%, cocaine 0.6% to 0.3%, opiates 0.5% to 0.3%, hallucinogens from 0.5% to 0.1% and inhalants from 0.5% to 0.2%.^5^ The increase in the prevalence of past 3-month drug use from 2008 to 2012 seems to be even higher among the youth (15–24 years) from 4.2% to 5.7%. This seems to show that the increase in any drug use is mainly attributed to an increase in cannabis use and a decrease in poly drug use (two or more drugs) from 0.7% in 2008 to 0.5% in 2012. The increase in the prevalence of cannabis use may be attributed to lower costs and better accessibility than the other drugs. One review^18^ indicates a global increase in substance use, including illicit drug use, in particular among young people, emphasising the importance of interventions. The use of *Whoonga* does not seem to be insignificant. In a qualitative study:

perceptions of whoonga suggest that it is highly addictive, contains ARVs (notably efavirenz), and poses a threat to the health and safety of those who use it, including increasing the risk of HIV infection.^19^

The use of amphetamine-type stimulants including *tik* and others had been as high as 2.2% in the Western Cape province in the 2008 survey,^5^ but was only 0.8% in the Western Cape in this survey, which may indicate some form of control as compared to previously.^20^ The increase in the use of drugs by injection from 0.3% in 2008 to 0.6% in 2012 is of concern. A study of 450 people who inject drugs (PWID) in South Africa found that they engaged in high HIV risk behaviour, including 49.0% having ‘used contaminated injecting equipment the last time they injected’.^21^

As found in previous studies,^5,6,7,22^ including in South Africa, the prevalence of drug use was significantly higher among men than women. Further, in agreement with some previous studies,^5,6^ this study found that younger age and being from the mixed race population group were associated with drug use. Unemployment was among men in bivariate analysis associated with drug use, as found in some previous studies.^5^

In agreement with previous reviews,^8,9,10,11,12^ this study found that hazardous or harmful or probable dependent alcohol use among both men and women, having had more than one sexual partner in the past 12 months among men, having been victim of violent crime among men and psychological distress (anxiety and depression) among women were associated with drug use. Drug use prevention and intervention have to include co-morbidity factors such as common mental disorders, alcohol use disorders and sexual risk behaviour.

## Limitations

This study was cross-sectional and no causative conclusions between independent variables and drug use can be drawn. The data on drug use were collected by self-report and may underreport the true consumption rate.^23^ Furthermore, the internal consistency of the ASSIST questionnaire for the drug use section was only moderate (0.61). Possible reasons for this may be that alcohol and tobacco use were not assessed as part of the ASSIST and an additional item (*Whoonga* use) had been added. Further, the ASSIST assesses methadone as an opioid, while technically it is a synthetic opioid.

## Conclusion

An increase of any illicit drug use prevalence rates was observed from 2008 to 2012 in South Africa. Prevention and intervention activities targeting illicit drug use, in particular in identified risk groups, need to be strengthened in South Africa.

## References

[1] United Nations Office on Drugs and Crime World drug report 2016. Sales No. E.16.XI.7. New Work: United Nations Publication; 2016.

[2] World Health Organization (WHO) Other psychoactive substances [homepage on the Internet]. [cited 2017 Jun 04]. Available from: http://www.who.int/substance_abuse/facts/psychoactives/en/

[3] Degenhardt L, Whiteford HA, Ferrari AJ, et al Global burden of disease attributable to illicit drug use and dependence: Findings from the Global Burden of Disease Study 2010. Lancet. 2013;382(9904):1564–1574. 10.1016/S0140-6736(13)61530-5 23993281

[4] Center for Behavioral Health Statistics and Quality Key substance use and mental health indicators in the United States: Results from the 2015 National Survey on Drug Use and Health [homepage on the Internet]. HHS Publication No. SMA 16-4984, NSDUH Series H-51 2016 [cited 2017 Jun 04]. Available from: http://www.samhsa.gov/data/

[5] Peltzer K, Ramlagan S Illicit drug use in South Africa: Findings from a 2008 national population-based survey. S Afr J Psychitry. 2010;16(1):8–15. 10.4102/sajpsychiatry.v16i1.230

[6] Van Heerden MS, Grimsrud AT, Seedat S, Myer L, Williams DR, Stein DJ Patterns of substance use in South Africa: Results from the South African Stress and Health study. S Afr Med J. 2009;99:358–366. 19588799 PMC3203645

[7] Peltzer K, Ramlagan S, Johnson BD, Phaswana-Mafuya N Illicit drug use and treatment in South Africa: A review. Subst Use Misuse. 2010;45(13):2221–2243. 10.3109/10826084.2010.481594 21039113 PMC3010753

[8] Conway KP, Swendsen J, Husky MM, He JP, Merikangas KR Association of lifetime mental disorders and subsequent alcohol and illicit drug use: Results from the National Comorbidity Survey-adolescent supplement. J Am Acad Child Adolesc Psychiatry. 2016;55(4):280–288. 10.1016/j.jaac.2016.01.006 27015718

[9] Lai HM, Cleary M, Sitharthan T, Hunt GE Prevalence of comorbid substance use, anxiety and mood disorders in epidemiological surveys, 1990–2014: A systematic review and meta-analysis. Drug Alcohol Depend. 2015;154:1–13. 10.1016/j.drugalcdep.2015.05.031 26072219

[10] Teesson M, Farrugia P, Mills K, Hall W, Baillie A Alcohol, tobacco, and prescription drugs: The relationship with illicit drugs in the treatment of substance users. Subst Use Misuse. 2012;47(8–9):963–971. 10.3109/10826084.2012.663283 22676566

[11] Meader N, King K, Moe-Byrne T, et al A systematic review on the clustering and co-occurrence of multiple risk behaviours. BMC Public Health. 2016;16:657 10.1186/s12889-016-3373-6 27473458 PMC4966774

[12] Walsh K, Resnick HS, Danielson CK, et al Patterns of drug and alcohol use associated with lifetime sexual revictimization and current posttraumatic stress disorder among three national samples of adolescent, college, and household-residing women. Addict Behav. 2014;39(3):684–689. 10.1016/j.addbeh.2013.12.006 24370205 PMC4018814

[13] Shisana O, Rehle T, Simbayi LC, et al South African national HIV prevalence, incidence and behaviour survey, 2012. Cape Town: HSRC Press; 2014.

[14] Humeniuk RE, Henry-Edwards S, Ali RL, Poznyak V, Monteiro M The Alcohol, Smoking and Substance Involvement Screening Test (ASSIST): Manual for use in primary care. Geneva: World Health Organization; 2010.

[15] Kessler RC, Barker PR, Colpe LJ, et al Screening for serious mental illness in the general population. Arch Gen Psychiatry. 2003;60(2):184e–189e. 10.1001/archpsyc.60.2.184 12578436

[16] Andersen LS, Grimsrud A, Myer L, Williams DR, Stein DJ, Seedat S The psychometric properties of the K10 and K6 scales in screening for mood and anxiety disorders in the South African Stress and Health study. Int J Methods Psychiatr Res. 2011;20(4):215–223. 10.1002/mpr.351 22113964 PMC6878543

[17] Babor TF, Higgins-Biddle JC, Saunders JB, Monteiro MG AUDIT: The Alcohol Use Disorders Identification Test Guidelines for use in primary care. Geneva, Switzerland: World Health Organization; 2001.

[18] Degenhardt L, Stockings E, Patton G, Hall WD, Lynskey M The increasing global health priority of substance use in young people. Lancet Psychiatry. 2016;3(3):251–264. 10.1016/S2215-0366(15)00508-8 26905480

[19] Grelotti DJ, Closson EF, Smit JA, et al Whoonga: Potential recreational use of HIV antiretroviral medication in South Africa. AIDS Behav. 2014;18(3):511–518. 10.1007/s10461-013-0575-0 23955659 PMC3926908

[20] Simbayi LC, Kalichman SC, Cain D, Cherry C, Henda N, Cloete A Methamphetamine use and sexual risks for HIV infection in Cape Town, South Africa. J Subst Use. 2006;11(4):291–300. 10.1080/14659890600625767

[21] Scheibe A, Makapela D, Brown B, et al HIV prevalence and risk among people who inject drugs in five South African cities. Int J Drug Policy. 2016;30:107–115. 10.1016/j.drugpo.2016.01.004 26860326

[22] Gureje O, Degenhardt L, Olley B, et al A descriptive epidemiology of substance use and substance use disorders in Nigeria during the early 21st century. Drug Alcohol Depend. 2007;91(1):1–9. 10.1016/j.drugalcdep.2007.04.010 17570618

[23] Gryczynski J, Schwartz RP, Mitchell SG, O’Grady KE, Ondersma SJ Hair drug testing and self-reported drug use among primary care patients with moderate-risk illicit drug use. Drug Alcohol Depend. 2014;141:44–50. 10.1016/j.drugalcdep.2014.05.001 24932945 PMC4080811

